# Comorbidity increases the risk of pulmonary tuberculosis: a nested case-control study using multi-source big data

**DOI:** 10.1186/s12890-023-02817-6

**Published:** 2024-01-11

**Authors:** Bao-Yu Wang, Ke Song, Hai-Tao Wang, Shan-Shan Wang, Wen-Jing Wang, Zhen-Wei Li, Wan-Yu Du, Fu-Zhong Xue, Lin Zhao, Wu-Chun Cao

**Affiliations:** 1 Institute of EcoHealth, School of Public Health, Cheeloo College of Medicine, Shandong University, Jinan, 250012 China; 2 Department of Epidemiology, School of Public Health, Cheeloo College of Medicine, Shandong University, Jinan, 250012 China; 3 Department of Biostatistics, School of Public Health, Cheeloo College of Medicine, Shandong University, 250012 Jinan, China; 4 Institute for Medical Dataology, School of Public Health, Cheeloo College of Medicine, Shandong University, Jinan, 250002 China; 5 State Key Laboratory of Pathogen and Biosecurity, Beijing Institute of Microbiology and Epidemiology, 20 Dongda Street, Fengtai District, Beijing, 100071 China

**Keywords:** Pulmonary Tuberculosis, Comorbidity, Multimorbidity, Prevention, Nested case-control study

## Abstract

**Background:**

Some medical conditions may increase the risk of developing pulmonary tuberculosis (PTB); however, no systematic study on PTB-associated comorbidities and comorbidity clusters has been undertaken.

**Methods:**

A nested case-control study was conducted from 2013 to 2017 using multi-source big data. We defined cases as patients with incident PTB, and we matched each case with four event-free controls using propensity score matching (PSM). Comorbidities diagnosed prior to PTB were defined with the International Classification of Diseases-10 (ICD-10). The longitudinal relationships between multimorbidity burden and PTB were analyzed using a generalized estimating equation. The associations between PTB and 30 comorbidities were examined using conditional logistic regression, and the comorbidity clusters were identified using network analysis.

**Results:**

A total of 4265 cases and 17,060 controls were enrolled during the study period. A total of 849 (19.91%) cases and 1141 (6.69%) controls were multimorbid before the index date. Having 1, 2, and ≥ 3 comorbidities was associated with an increased risk of PTB (aOR 2.85–5.16). Fourteen out of thirty comorbidities were significantly associated with PTB (aOR 1.28–7.27), and the associations differed by sex and age. Network analysis identified three major clusters, mainly in the respiratory, circulatory, and endocrine/metabolic systems, in PTB cases.

**Conclusions:**

Certain comorbidities involving multiple systems may significantly increase the risk of PTB. Enhanced awareness and surveillance of comorbidity are warranted to ensure early prevention and timely control of PTB.

**Supplementary Information:**

The online version contains supplementary material available at 10.1186/s12890-023-02817-6.

## Introduction

Pulmonary tuberculosis (PTB), accounting for almost 50% of all new tuberculosis (TB) cases reported globally in 2021, continues to pose a serious threat to global health due to its high morbidity and mortality [1]. Some immunocompromised states, including those produced by aging, chronic diseases (such as HIV/AIDS and diabetes mellitus), and immunosuppressive therapy, may contribute to the development of PTB [2, 3]. However, few studies have systematically analyzed the comorbidities associated with PTB and their potential clusters. Additionally, previous studies have mostly focused on a single medical condition through cross-sectional or cohort studies. Nested case-control studies allow us to simultaneously investigate the causative role of multiple risk factors in the development of an outcome. The exposure data are measured prior to the outcome occurring, and the design provides an efficient way to identify causal relationships [4]. Multi-source healthcare big data, involving all communicable and non-communicable diseases of an individual that occur during the study period [5], give us the chance to recognize the associations between PTB and multiple diseases, as well as the complex interactions among comorbidities.

Although China has the third highest TB burden in the world, with 7.4% of the incident cases [1], the influence of comorbidities on PTB has not yet been fully described. In the present study, we carried out a nested case-control study based on the Shandong Multi-Center Healthcare Big Data Platform (SMCHBDP). We aimed to investigate the relationships between PTB and prevalence of comorbidity, recognize the risk factors favoring the development of PTB, elaborate the comorbidity clusters in PTB patients, and thus devise targeted control efforts to fight against PTB in China.

## Materials and methods

### Study design and population

We conducted a nested case-control study using the Shandong Multi-Center Healthcare Big Data Platform (SMCHBDP) [5, 6]. Multi-stage sampling was used to select the potentially eligible participants in rural and urban areas of Shandong Province, based on their identity numbers registered on the SMCHBDP [6]. Multiple sources of health-related data, including electronic medical health records, basic public health records, and resident medical insurance payment systems, were integrated and linked using unique identity numbers. A total of five million participants in Shandong Province, China, were involved. Each eligible participant’s information concerning demographic characteristics (age, sex, and place of residence) and medical history (diagnoses, prescriptions, and past diseases) was extracted. Then, participants registered on the SMCHBDP from 1 January 2013 to 31 December 2017 were included, and those with duplicate or incorrect identity numbers were excluded. Furthermore, participants with evident outliers or logical errors in case information, making it impossible to link disease visit information to specific individuals, were also excluded. Additionally, since some participants were registered at multiple medical institutions, we used the earliest registration date as the personal finishing baseline filling date in the SMCHBDP. Finally, a total of 4,057,987 participants were included in the present study from 1 January 2013 to 31 December 2017. The follow-up time started at the date of the enrollment of participants (finishing baseline filling date) and ended at the time of incident PTB, death, or on 31 December 2017. This study was approved by the Ethics Committee of the School of Public Health, Shandong University, China. All individual information has been anonymized and informed consent was waived owing to the retrospective nature of the study.

### Definitions of cases and controls

**Fig. 1:**
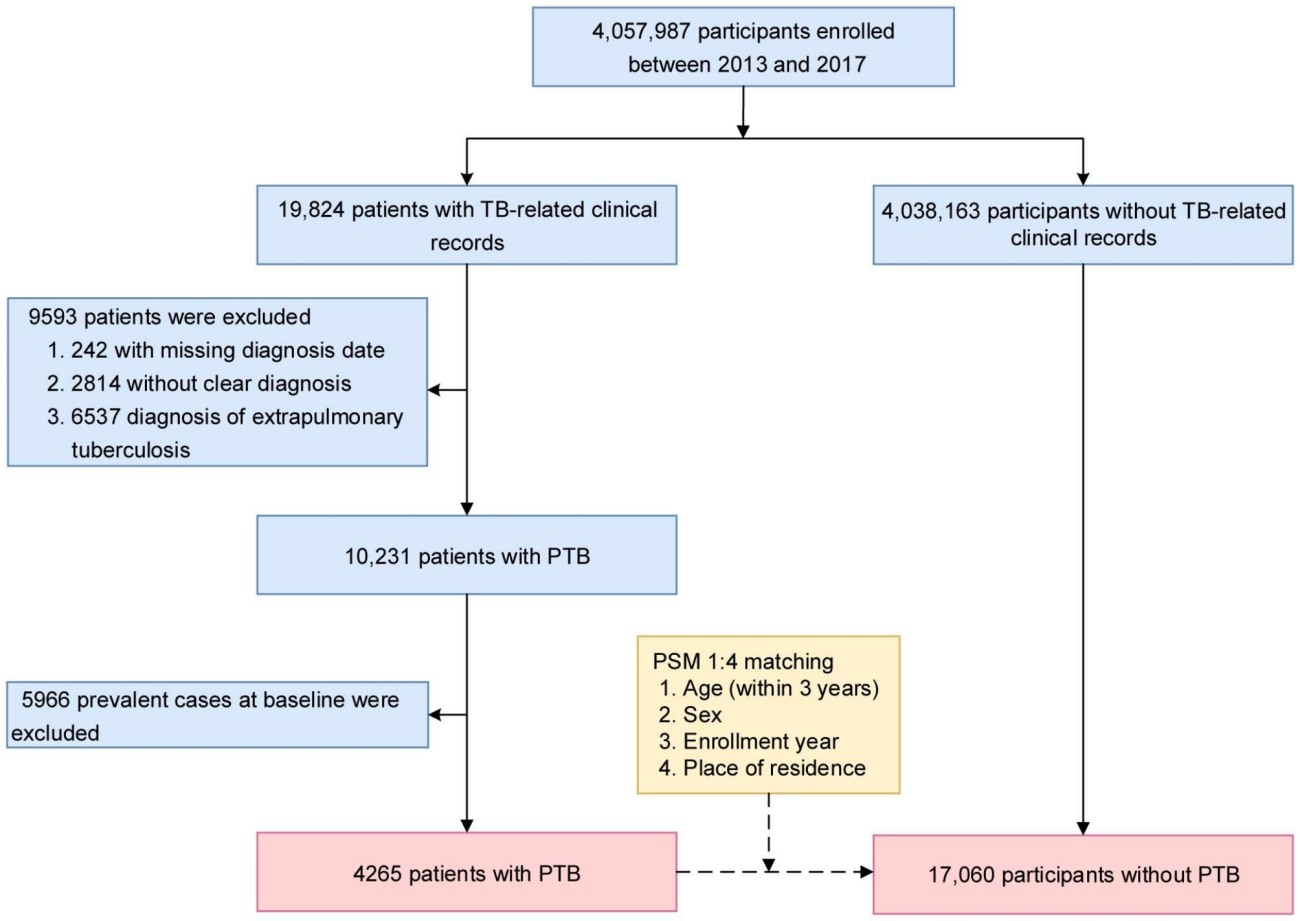
Study flowchart for the selection of PTB cases and controls. PTB, pulmonary tuberculosis

Confirmed PTB cases, based on symptoms, chest X-rays, and positive sputum smears or cultures, were diagnosed by qualified physicians according to the National Diagnostic Criteria for Pulmonary Tuberculosis [7] and the 10th revision of the International Statistical Classification of Diseases (ICD-10). The date of initial diagnosis for PTB served as the index date. There were a total of 19,824 patients with TB-related clinical records in the database. We then excluded the patients with missing diagnosis date (n = 242), without clear diagnosis (n = 2814), diagnosis of extrapulmonary tuberculosis (n = 6537), and prevalent PTB cases at baseline (n = 5966). For each case, four event-free controls (participants without TB-related clinical records) alive on the index date were identified using the propensity score matching (PSM) method [8], matching age (within 3 years), sex, enrollment year, and place of residence. In total, 4265 PTB cases and 17,060 controls were enrolled. The sampling process is depicted in Fig. 1.

### Comorbidity

Comorbidity was defined as any distinct medical condition that existed before the occurrence of PTB. Diseases that met the following criteria were included in further analysis: (1) having a potential impact on the development of tuberculosis, (2) having a prevalence of > 0.1% in cases, and (3) having at least two diagnostic records. We finally selected 30 clinically important comorbidities in the present study, and all these diseases were defined according to the ICD-10 (Table S1). The date of the first diagnostic record of diseases served as the onset date. Multimorbidity was defined as the co-occurrence of at least 2 conditions from the above 30 comorbidities within a person. In addition, the Charlson-Deyo Comorbidity Index (CCI) was used to evaluate the burden of multimorbidity [9].

### Statistical analysis

The normality of continuous data was tested using the Kolmogorov-Smirnov test. Skewed data were expressed as median (interquartile range, IQR) and compared using the Kruskal-Wallis test. Categorical variables were shown as frequency (%), and the differences were tested using a chi-squared test. The generalized estimating equation (GEE) was utilized to analyze the longitudinal relationships between multimorbidity burden and PTB. Conditional logistic regression analysis was used to examine the association between PTB and 30 comorbidities in the entire study population and in subgroups stratified by age (0–44 years, 45–64 years, and ≥ 65 years) and sex. Diseases with statistical significance in multifactorial conditional logistic regression (adjusting for age at index date, sex, and all comorbidities) were included to create an undirected weighted comorbidity network. The co-occurrence intensity between different diseases was measured using the observed-to-expected ratio (OER), and comorbidity clusters were recognized using the community detection algorithm Louvain [10]. The false discovery rate (FDR) was controlled for multiple testing with the Benjamini-Hochberg procedure [11]. All statistical analyses were performed using R software (version 4.1.2, R Foundation for Statistical Computing, Vienna, Austria), and a *P* value < 0.05 was considered statistically significant.

## Results

### Demographic characteristics and multimorbidity

The detailed information on characteristics of participants is displayed in Table 1. The median age of both cases and controls was 46 years (IQR 27–62), and 63.26% of them were male. There were no significant differences in matching variables between the two groups (*P* > 0.05). The median time from entrance into the cohort to the index date for the PTB and control groups was 2.54 years (IQR 1.26–3.68) and 2.58 years (IQR 1.30–3.72), respectively. Compared with the control group, PTB cases had a higher proportion of having comorbidities during the study period. In total, 849 (19.91%) cases and 1141 (6.69%) controls were multimorbid. The prevalence of multimorbidity increased with age, and the sex-specific prevalence was higher in female than in male in all age groups (Table S2). According to the CCI score, cases with PTB tended to have a moderate (20.42% vs. 6.73%) or high (4.92% vs. 1.27%) burden of multimorbidity compared with controls (Table 1).

**Table 1:** Characteristics of patients with PTB and matched controls

	Cases (n = 4265)	Controls (n = 17,060)	*p-Value
Age at enrollment, years			0.934
Median (IQR)	46 (27, 62)	46 (27, 62)	
Sex, n (%)			1.000
Female	1567 (36.74)	6268 (36.74)	
Male	2698 (63.26)	10,792 (63.26)	
Place of residence, n (%)			1.000
Rural	1607 (37.68)	6426 (37.67)	
Urban	2658 (62.32)	10,634 (62.33)	
Southern Shandong ^†^	1192 (27.95)	4772 (27.97)	
Northern Shandong ^‡^	976 (22.88)	3902 (22.87)	
Central Shandong ^§^	1009 (23.66)	4034 (23.65)	
Jiaodong Peninsula ^¶^	1088 (25.51)	4352 (25.51)	
Enrollment year, n (%)			1.000
2013	3555 (83.35)	14,220 (83.35)	
2014	340 (7.97)	1360 (7.97)	
2015	266 (6.24)	1062 (6.23)	
2016	79 (1.85)	318 (1.86)	
2017	25 (0.59)	100 (0.59)	
Time from entry to index date, years			0.100
Median (IQR)	2.54 (1.26, 3.68)	2.58 (1.30, 3.72)	
Number of comorbidities, n (%)			< 0.001
None	2741 (64.26)	14,409 (84.46)	
1	675 (15.83)	1510 (8.85)	
2	351 (8.23)	600 (3.52)	
≥ 3	498 (11.68)	541 (3.17)	
CCI score			< 0.001
Low, CCI = 0	3184 (74.66)	15,695 (92.00)	
Moderate, CCI = 1–2	871 (20.42)	1149 (6.73)	
High, CCI > 2	210 (4.92)	216 (1.27)	

IQR, interquartile range; PTB, pulmonary tuberculosis; CCI, Charlson–Deyo Comorbidity Index. ^†^ Southern Shandong: Jining, Heze, Zaozhuang, Linyi, and Rizhao; ^‡^ Northern Shandong: Liaocheng, Dezhou, Binzhou, and Dongying; ^§^ Central Shandong: Jinan, Zibo, Weifang, and Taian; ^¶^ Jiaodong Peninsula: Qingdao, Yantai, and Weihai. * *p* < 0.05 was considered statistically significant

### Associations between PTB and multimorbidity

**Fig. 2:**
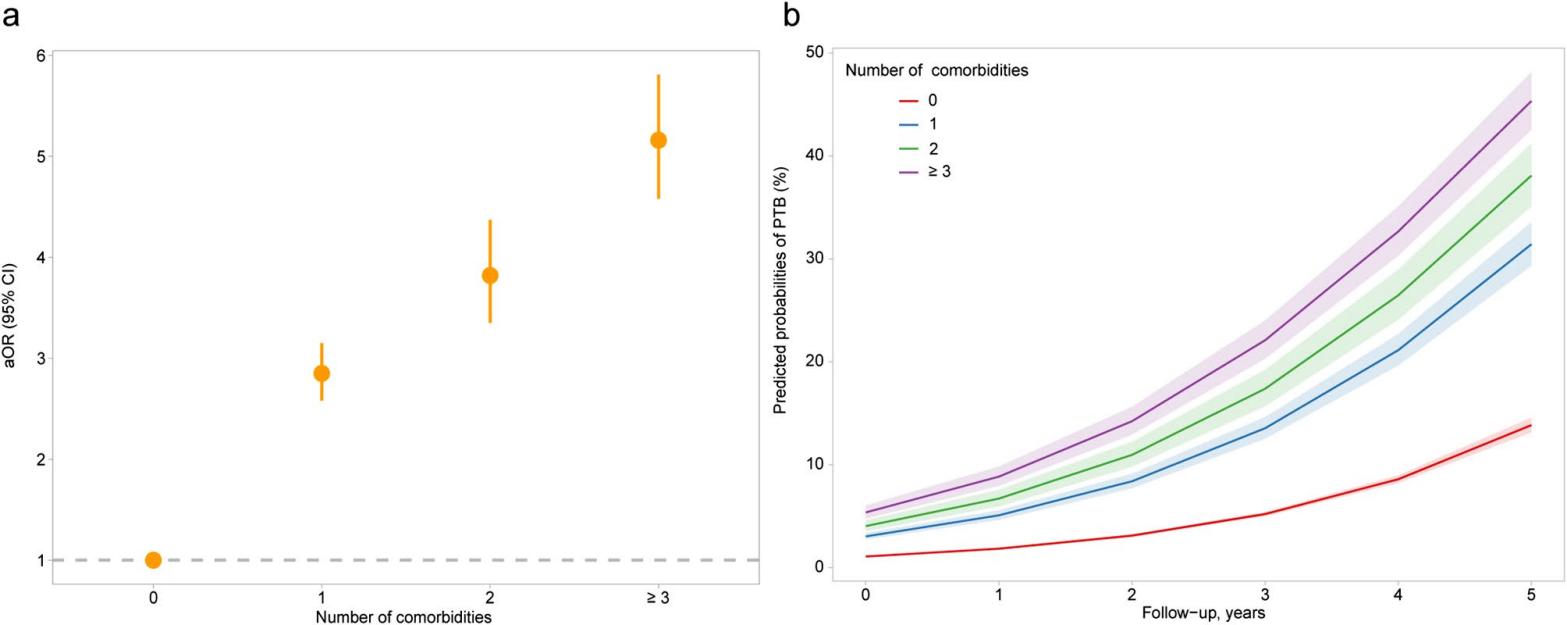
Associations between PTB and multimorbidity burden. (**a**) Association between multimorbidity burden and risk of PTB is expressed as odds ratios. Age, sex, and year of follow-up were adjusted for in the generalized estimating equation, with no comorbidity used as the reference (aOR = 1); (**b**) longitudinal associations between multimorbidity burden and predicted probabilities of PTB were estimated using a generalized estimating equation. aOR, adjusted odds ratio; PTB, pulmonary tuberculosis

Compared with the controls, cases were more likely to have comorbidities at baseline and during follow-up (Fig. S1). The risk of PTB increased with the number of comorbidities from an adjusted odds ratio (aOR) of 2.85 (95% CI 2.58–3.15) for individuals with one comorbidity to 5.16 (95% CI 4.58–5.81) for those with three or more comorbidities (Fig. 2a). The predicted probabilities of PTB considerably increased over time among all individuals, and this trend was more obvious in individuals with multimorbidity (Fig. 2b).

### Comorbidity and risk of PTB

All comorbidities were more prevalent in patients with PTB compared with matched controls (Table 2). The top five diseases in PTB cases were hypertensive diseases (14.40%), chronic lower respiratory diseases (10.83%), chronic ischemic heart disease (9.52%), diabetes mellitus (9.21%), and cerebrovascular diseases (6.80%). Fourteen comorbidities were associated with an increased risk of PTB after adjusting for potential confounding variables. The two comorbidities with the strongest associations were interstitial lung diseases (aOR 7.27, 95% CI 3.61–14.64) and chronic lower respiratory diseases (aOR 7.25, 95% CI 5.98–8.80). Individuals with comorbidities, including connective tissue diseases, inflammatory diseases of the central nervous system, cancer, inflammatory diseases of female pelvic organs, chronic liver diseases, diabetes mellitus, and disorders of the peripheral nervous system, were two to three times as likely to have PTB compared with those without comorbidities. The risk of PTB for individuals with cerebrovascular diseases, disorders of the thyroid gland, noninfective enteritis and colitis, chronic ischemic heart disease, and metabolic disorders was slightly but significantly higher with an aOR of 1–2.

Subgroup analyses by sex and age at index date were carried out. The aOR was significantly higher in female with interstitial lung diseases (aOR 19.40, 95% CI 4.03–93.37), chronic lower respiratory diseases (aOR 9.58, 95% CI 6.60–13.92), and connective tissue diseases (aOR 6.00, 95% CI 2.21–16.29) than in male (Tables S3 and S4). Cardiac arrhythmias (aOR 6.63, 95% CI 1.62–27.17) and Intervertebral disc disorders (aOR 3.61, 95% CI 1.57–8.28) were significantly associated with PTB in individuals aged 44 years and younger, while anemias increased the risk of developing PTB within the group of those aged 45–64 years (aOR 5.68, 95% CI 1.92–16.78) (Tables S5 and S6). The two comorbidities with the strongest associations in patients over 65 were chronic lower respiratory diseases (aOR 6.67, 95% CI 5.16–8.62) and interstitial lung diseases (aOR 5.76, 95% CI 2.42–13.71) (Table S7).

**Table 2:** Associations between PTB and comorbidities

Comorbidities	Cases n (%)	Controls n (%)	Unadjusted		Adjusted ^a^	
			OR (95% CI)	** *p-*Value	OR (95% CI)	** *p-*Value
Neoplasms						
Cancer	144 (3.38)	137 (0.80)	4.78 (3.72, 6.14)	< 0.001	3.15 (2.35, 4.21)	< 0.001
Diseases of the blood and blood-forming organs						
Anemias	67 (1.57)	58 (0.34)	4.88 (3.40, 7.00)	< 0.001	1.56 (1.00, 2.43)	0.090
Endocrine/metabolic diseases						
Disorders of the thyroid gland	66 (1.55)	63 (0.37)	4.27 (3.01, 6.05)	< 0.001	1.89 (1.24, 2.90)	0.010
Diabetes mellitus	393 (9.21)	605 (3.55)	3.00 (2.61, 3.45)	< 0.001	2.07 (1.76, 2.44)	< 0.001
Metabolic disorders	140 (3.28)	122 (0.72)	4.94 (3.84, 6.35)	< 0.001	1.56 (1.14, 2.14)	0.018
Mental and behavioral disorders						
Mental disorders	37 (0.87)	57 (0.33)	2.63 (1.73, 4.00)	< 0.001	0.89 (0.51, 1.53)	0.666
Nervous system diseases						
Inflammatory diseases of the central nervous system	52 (1.22)	31 (0.18)	6.71 (4.3, 10.47)	< 0.001	3.26 (1.84, 5.77)	< 0.001
Epilepsy	12 (0.28)	18 (0.11)	2.67 (1.28, 5.54)	0.008	1.49 (0.66, 3.36)	0.489
Disorders of the peripheral nervous system	49 (1.15)	34 (0.20)	6.26 (3.97, 9.89)	< 0.001	2.07 (1.17, 3.66)	0.032
Diseases of the eye and adnexa						
Cataract	58 (1.36)	77 (0.45)	3.11 (2.19, 4.39)	< 0.001	1.40 (0.90, 2.17)	0.222
Circulatory system diseases						
Valvular heart diseases	14 (0.33)	20 (0.12)	2.86 (1.43, 5.72)	0.003	0.77 (0.33, 1.78)	0.617
Hypertensive diseases	614 (14.40)	1538 (9.02)	1.94 (1.73, 2.17)	< 0.001	1.10 (0.95, 1.27)	0.309
Chronic ischemic heart disease	406 (9.52)	653 (3.83)	3.13 (2.71, 3.62)	< 0.001	1.58 (1.31, 1.90)	< 0.001
Pulmonary heart disease and diseases of pulmonary circulation	55 (1.29)	32 (0.19)	7.20 (4.61, 11.24)	< 0.001	1.84 (1.06, 3.19)	0.062
Cardiac arrhythmias	87 (2.04)	101 (0.59)	3.82 (2.82, 5.18)	< 0.001	1.12 (0.76, 1.66)	0.617
Cerebrovascular diseases	290 (6.80)	533 (3.12)	2.54 (2.16, 2.98)	< 0.001	1.28 (1.05, 1.57)	0.035
Respiratory system diseases						
Chronic lower respiratory diseases	462 (10.83)	224 (1.31)	10.55 (8.82, 12.62)	< 0.001	7.25 (5.98, 8.80)	< 0.001
Interstitial lung diseases	53 (1.24)	13 (0.08)	16.31 (8.89, 29.91)	< 0.001	7.27 (3.61, 14.64)	< 0.001
Digestive system diseases						
Ulcer diseases	21 (0.49)	43 (0.25)	2.01 (1.18, 3.43)	0.011	0.71 (0.36, 1.40)	0.479
Noninfective enteritis and colitis	53 (1.24)	55 (0.32)	3.89 (2.66, 5.69)	< 0.001	1.82 (1.13, 2.93)	0.032
Chronic liver diseases	22 (0.52)	22 (0.13)	4.22 (2.29, 7.75)	< 0.001	2.28 (1.15, 4.55)	0.041
Diseases of the skin and subcutaneous tissue						
Dermatitis and eczema	25 (0.59)	35 (0.21)	2.89 (1.72, 4.85)	< 0.001	1.31 (0.67, 2.57)	0.590
Papulosquamous disorders	12 (0.28)	16 (0.09)	3.00 (1.42, 6.34)	0.004	1.35 (0.55, 3.35)	0.617
Musculoskeletal/connective tissue diseases						
Connective tissue diseases	35 (0.82)	19 (0.11)	7.37 (4.22, 12.88)	< 0.001	3.62 (1.87, 7.00)	< 0.001
Intervertebral disc disorders	77 (1.81)	134 (0.79)	2.41 (1.80, 3.22)	< 0.001	1.12 (0.78, 1.60)	0.617
Osteoporosis	14 (0.33)	16 (0.09)	3.62 (1.74, 7.51)	0.001	1.38 (0.57, 3.33)	0.617
Genitourinary system diseases						
Renal diseases	56 (1.31)	83 (0.49)	2.75 (1.95, 3.88)	< 0.001	1.12 (0.73, 1.72)	0.633
Disorders of prostate	58 (1.36)	99 (0.58)	2.60 (1.84, 3.69)	< 0.001	0.91 (0.58, 1.41)	0.666
Disorders of breast	16 (0.38)	24 (0.14)	2.71 (1.43, 5.14)	0.002	1.76 (0.86, 3.59)	0.214
Inflammatory diseases of female pelvic organs	61 (1.43)	82 (0.48)	3.45 (2.40, 4.95)	< 0.001	2.51 (1.69, 3.72)	< 0.001

^a^ Adjusted odds ratio (aOR) for age at index date, sex, and all comorbidities. ** *p* < 0.05 adjusted for multiple testing using FDR. FDR, false discovery rate

### Comorbidity network

**Fig. 3:**
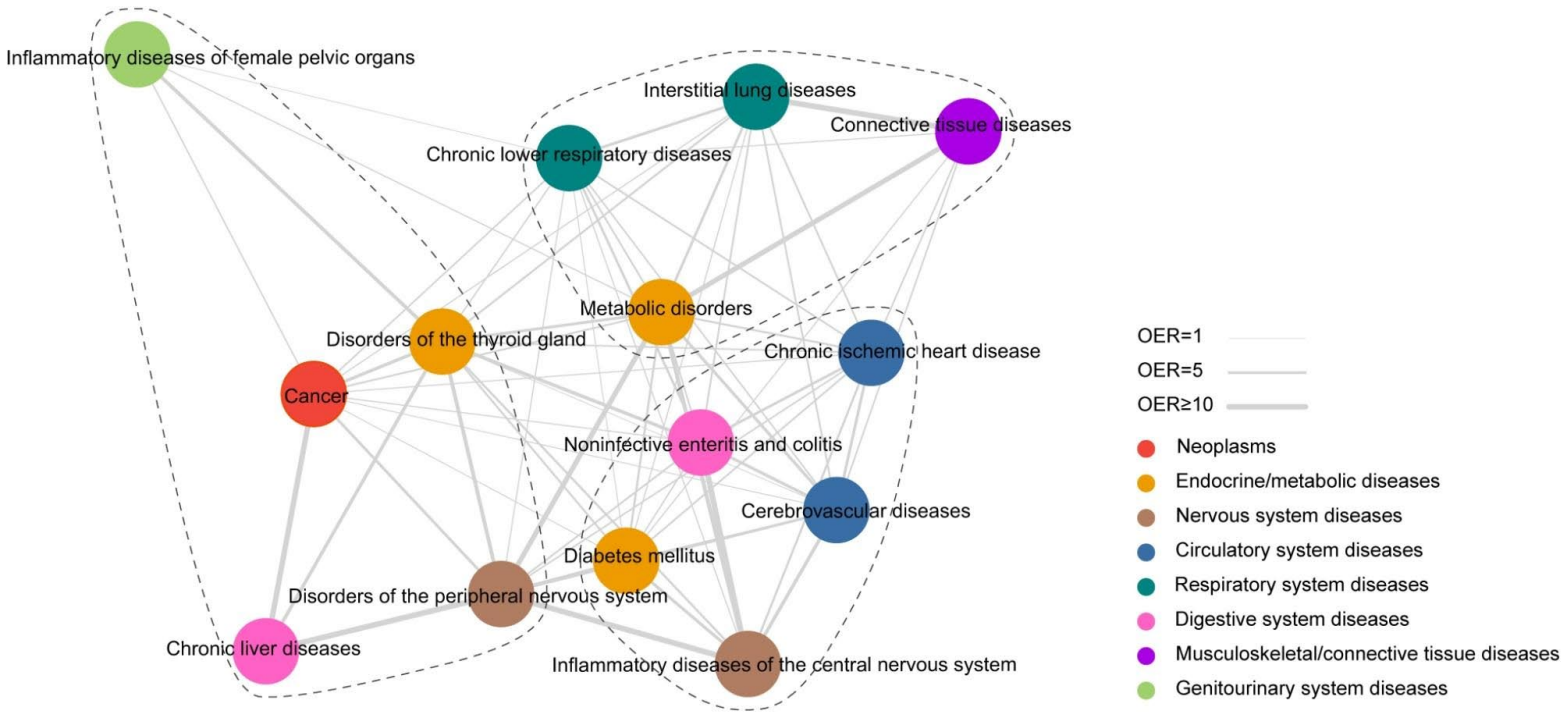
Comorbidity network in patients with PTB. Each node represents a disease. Color of each node indicates the disease category. Width of edge represents the strength of comorbidity association measured using observed-to-expected ratio. Three clusters of the comorbidity network are represented with dashed lines. PTB, pulmonary tuberculosis. OER, observed-to-expected ratio

Figure 3 depicts the network of the fourteen comorbidities significantly associated with PTB. Based on the degree centrality values, chronic lower respiratory diseases, diabetes mellitus, chronic ischemic heart disease, cerebrovascular diseases, and cancer were key conditions in the PTB comorbidity network (Table S8). The strongest links occurred among endocrine/metabolic, digestive, respiratory, nervous, and musculoskeletal/connective tissue diseases. For example, noninfective enteritis and colitis co-occurred with inflammatory diseases of the central nervous system, interstitial lung diseases co-occurred with connective tissue diseases, and diabetes mellitus co-occurred with disorders of the peripheral nervous system (Fig. 3) (Table S9). Modularity analysis revealed three clusters: (a) chronic lower respiratory diseases, interstitial lung diseases, connective tissue diseases, and metabolic disorders; (b) chronic ischemic heart disease, diabetes mellitus, cerebrovascular diseases, noninfective enteritis and colitis, and inflammatory diseases of the central nervous system; (c) cancer, disorders of the thyroid gland, disorders of the peripheral nervous system, inflammatory diseases of female pelvic organs, and chronic liver diseases (Fig. 3).

## Discussion

In this nested case-control study, a higher proportion of PTB cases were multimorbid before the index date. The increased number of comorbidities might lead to a higher risk of developing PTB. Among 30 comorbidities, 14 were found to be significantly positively associated with PTB, ranging from an aOR of 1.28 (cerebrovascular diseases) to 7.27 (interstitial lung diseases). Three clusters of comorbidities, affecting the respiratory, circulatory, and endocrine/metabolic systems, were observed. Our findings shed new light on the high-risk population of PTB and provide a scientific basis for targeted prevention policies for the disease.

Strong links between TB and other communicable diseases or noncommunicable diseases (NCDs) have been found in many countries, especially in developing countries, resulting in a “double burden of disease” [12]. Patients with PTB, which is considered the most common manifestation of TB, have concurrent various comorbidities in clinical practice. The present study found that 19.91% of PTB cases had at least two comorbidities before the index date, and the multimorbidity burden was particularly high in female and older cases. Both multimorbidity and PTB were associated with high mortality, declined physical function, and increased socioeconomic and medical burden [13]. A cross-sectional survey conducted in 48 low- and middle-income countries showed that people with 1, 2, 3, 4, and ≥ 5 noncommunicable diseases had from 2.61 to 19.89 times higher odds for TB [14]. A multicenter observational study indicated that having one or more comorbidities that weaken the immune response may cause patients to become pre-disposed to developing PTB [15]. Our nested case-control study in Shandong Province, China, found that the risk of PTB tended to increase with an increasing number of comorbidities, which was consistent with the previous reports. A better understanding of the associations between multimorbidity, comorbidities, and PTB is vital for reducing the double burden of diseases worldwide, in particular in developing countries.

After adjusting for age, sex, and other comorbidities, 14 comorbidities across 8 systems were significantly associated with the increasing risk of PTB, with odds ratios ranging from 1.28 to 7.27 in this study. Previous studies have demonstrated that diabetes mellitus was associated with an increased risk of PTB [16, 17]. The data here showed that individuals with diabetes mellitus were twice as likely to have PTB compared with those without diabetes mellitus (aOR 2.07, 95% CI 1.76–2.44). The morbidity and mortality of PTB and diabetes mellitus remain high, especially when these two diseases occur together [18]. Our findings strongly suggest that TB control measures should be integrated with diabetes mellitus control programs. We also demonstrated that the risk of PTB among patients with connective tissue diseases was significantly high (aOR 3.62, 95% CI 1.87–7.00). Previous studies have shown that patients with connective tissue diseases, such as rheumatoid arthritis [19, 20] and systemic lupus erythematosus [21], were at high risk for TB. The possible reasons for this may be due to the defective immunity resulting from the diseases or associated immunosuppressant therapy. These results indicate that healthcare workers need to be vigilant about the development of PTB among patients with connective tissue diseases. A review article on host-directed therapy suggests that the occurrence of NCDs could indicate a need for active TB screening for its early detection of TB and treatment outcomes improvement. And TB diagnosis provides a chance for clinicians to screen for NCDs like diabetes mellitus [22].

Globally, the associations between a history of chronic respiratory diseases and the presence of TB are inconsistent in different countries. One systematic review suggested an increased risk of TB in people with chronic obstructive pulmonary disease (COPD) in high-income countries [23]. In low- and middle-income countries with a high incidence of TB, information on the associations between TB and chronic respiratory diseases was limited. A Chinese cohort study involving 23,594 COPD cases and 47,188 control subjects revealed that COPD was an independent risk factor for developing PTB [24]. A study in India reported a significantly increased risk of TB in patients with asthma (adjusted odds ratio 2.5, 95% CI 1.8–3.7), while an inverse association was found in three countries in west Africa [25, 26]. Our results indicated the significant association between PTB and chronic lower respiratory diseases (aOR 7.25, 95% CI 5.98–8.80) after adjustment for age, sex, and other comorbidities. The possible reasons for such differences might include different Bacillus Calmette–Guérin (BCG) vaccination coverage, genetic and environmental factors among ethnic groups, and sample sizes [23]. These results suggested that the risk of PTB development in patients with chronic lung diseases needs further investigation. Additionally, we confirmed that patients with interstitial lung diseases had 7.27 times higher odds of developing PTB (aOR 7.27, 95% CI 3.61–14.64), which is consistent with the results of previous studies [27].

Previous studies have mainly focused on the impact of respiratory and endocrine/metabolic diseases on PTB development; the relationships between the comorbidities of other systems and PTB have received less attention. Big data derived from multiple sources made it possible to identify other potential risk factors of PTB. With regard to circulatory system diseases, Chidambaram et al. reported tuberculosis infection has been associated with acute myocardial infarction and atherothrombotic stroke [28]. Our study found that patients with chronic ischemic heart disease (aOR 1.58, 95% CI 1.31–1.90) and cerebrovascular diseases (aOR 1.28, 95% CI 1.05–1.57) had a higher risk of PTB. The possible reason for this association might be systemic inflammation and its mediation of chronic diseases [28]. For digestive system diseases, we found a strong association between noninfective enteritis and colitis and PTB after adjusting for confounding factors. The association between PTB and inflammatory bowel disease (IBD) was also reported in a recent study. Krusinski et al. found that biological agents for IBD may increase the susceptibility to TB [29]. Additionally, we found that some comorbidities demonstrated age discrepancies. Individuals with cardiac arrhythmias (aOR 6.63, 95% CI 1.62–27.17) or intervertebral disc disorders (aOR 3.61 95% CI 1.57–8.28) have a higher risk of PTB in the age group of 0–45 years. It is noted that these two diseases have not been considered as risk factors for PTB before. Anemias (aOR 5.68, 95% CI 1.92–16.78), a strong predictor of TB [30], had a significant association with PTB within the group of those aged 45–65 years in our study. Therefore, the increased risk of PTB among individuals with these conditions should be addressed.

To the best of our knowledge, this is the first study to systematically investigate the comorbidities and their complex associations in patients with PTB in China. Our results showed that chronic lower respiratory diseases, diabetes mellitus, chronic ischemic heart disease, cerebrovascular diseases, and cancer were more closely related to other diseases in the comorbidity network and contributed to a high risk of PTB. Some links between comorbidities should be emphasized, such as that between interstitial lung diseases and connective tissue diseases. Previous studies indicated that all patients with connective tissue diseases are at risk of interstitial lung diseases, and some connective tissue diseases (e.g., systemic sclerosis, antisynthetase syndrome, and rheumatoid arthritis) are more likely to be associated with interstitial lung diseases [31]. Modularity analysis in our study demonstrated three clusters, including diseases in the respiratory, circulatory, and endocrine/metabolic systems. Highly aggregated comorbidities in PTB patients may be the result of sharing the same gene or pathway of protein action or having similar risk factors or patterns of disease trajectory [32]. For instance, chronic ischemic heart disease, cerebrovascular diseases, and diabetes mellitus were classified into the same cluster. These are all complex, system-level diseases affecting many physiological and biomolecular processes; therefore, they co-occur with each other [33]. A recent network analysis showed that 86 key regulators were enriched by diverse biological processes and pathways, possibly connecting TB and NCDs, such as diabetes mellitus and cardiovascular diseases [34]. Analyzing comorbidity clusters may help us better understand associations between diseases rather than viewing them as isolated illnesses. Therefore, giving priority to controlling common risk factors in each cluster would be more efficient to reduce the comorbid burden with limited health resources, especially in high TB burden countries.

With the rapid development of computer science and information technology, healthcare big data provide a new approach to discover previously unappreciated relationships between communicable and non-communicable diseases. This nested case-control study using the SMCHBDP showed that having multiple comorbidities may increase the risk of developing PTB, and highlighted the complex interactions among comorbidities. Compared with traditional surveillance system, multi-source healthcare big data involved all medical conditions of an individual that occured during the study period. Additionally, the design of nested case-control study minimized selection bias and recall bias, making the causal association between PTB and comorbidities inferred from our study more reliable. Our research findings will enable healthcare workers to detect and prevent PTB at an early stage in populations with specific comorbidities. Due to the population-based nature of our study, we believe the results can be applied to other cities with comparable healthcare systems and accessibility to basic medical treatment. Future studies are required to understand the prevalence and distribution patterns of PTB comorbidity in the community.

This study has two main limitations. First, the lack of information on individual risk factors, including smoking habits, physical activities, socioeconomic status, drug resistance, and treatment outcome, might have affected the associations. Nonetheless, the most important variables influencing PTB development, including age, sex, and other comorbidities, were adjusted for in our analysis. Second, the sample size of PTB cases and some comorbidities was relatively small, and the follow-up time was relatively short. Therefore, this study might be underpowered. However, we still found associations between PTB and fourteen comorbidities. Future studies with a larger sample and a longer observation period will be needed to confirm the findings. Additionally, the design of nested case-control study minimized selection bias and recall bias. The causality inferred from the associations between PTB and comorbidities in our study could be more reliable.

## Conclusions

In conclusion, the risk of PTB increased incrementally for each additional comorbidity from an aOR of 2.85 for one comorbidity to 5.16 for ≥ 3 comorbidities. Fourteen comorbidities, such as interstitial lung diseases, chronic lower respiratory diseases, and diabetes mellitus, were associated with an increased risk of developing PTB. Diseases of respiratory, circulatory, and endocrine/metabolic systems tend to co-occur and cluster with each other. Enhanced awareness and surveillance of comorbidity are warranted, especially in countries with a high tuberculosis burden. Authorities and healthcare workers should adopt inclusive strategies that take more diseases and risk factors into account to ensure early prevention and timely control of PTB.

### Electronic supplementary material

Below is the link to the electronic supplementary material.

Supplementary Material 1

## Data Availability

The datasets generated and/or analyzed during the current study are not publicly available due individual privacy of patients could be compromised, but are available from the corresponding author on reasonable request.

## Abbreviations

TB: Tuberculosis
PTB: Pulmonary Tuberculosis
PSM: Propensity score matching
ICD-10: International Classification of Diseases-10
aOR: Adjusted odds ratio
HIV: Human Immunodeficiency Virus
AIDS: Acquired Immune Deficiency Syndrome
SMCHBDP: Shandong Multicenter Healthcare Big Data Platform
CCI: Charlson-Deyo Comorbidity Index
IQR: Interquartile range
CI: Confidence interval
GEE: Generalized estimating equation
OER: Observed-to-expected ratio
FDR: False discovery rate
NCDs: Noncommunicable diseases
COPD: Chronic obstructive pulmonary disease
BCG: Bacillus Calmette-Guérin
IBD: Inflammatory bowel disease
