# Supplementary material for: Comorbidity increases the risk of pulmonary tuberculosis: a nested case-control study using multi-source big data
Source: BMC Pulm Med. 2024 Jan 11;24:29. doi: 10.1186/s12890-023-02817-6 (PMC10782630; doi:10.1186/s12890-023-02817-6)
Supplement: Supplementary file 1 — Supplementary Material 1 [file 12890_2023_2817_MOESM1_ESM.docx]

**Supplemental Material**

Comorbidity increases the risk of pulmonary tuberculosis: a nested case-control study using multi-source big data

Bao-Yu Wang ^1,2^, Ke Song ^1,2^, Hai-Tao Wang ^2^, Shan-Shan Wang ^1,2^, Wen-Jing Wang ^1,2^, Zhen-Wei Li ^1^, Wan-Yu Du ^1^, Fu-Zhong Xue ^3,4^, Lin Zhao ^1,2,^* and Wu-Chun Cao ^1,5,^*

^1^ Institute of EcoHealth, School of Public Health, Cheeloo College of Medicine, Shandong University, Jinan 250012, China.

^2^ Department of Epidemiology, School of Public Health, Cheeloo College of Medicine, Shandong University, Jinan 250012, China.

^3^ Department of Biostatistics, School of Public Health, Cheeloo College of Medicine, Shandong University, Jinan 250012, China.

^4^ Institute for Medical Dataology, School of Public Health, Cheeloo College of Medicine, Shandong University, Jinan 250002, China.

^5^ State Key Laboratory of Pathogen and Biosecurity, Beijing Institute of Microbiology and Epidemiology, Beijing 100071, China.

* Corresponding authors:

Wu-Chun Cao, State Key Laboratory of Pathogen and Biosecurity, Beijing Institute of Microbiology and Epidemiology, 20 Dongda Street, Fengtai District, Beijing 100071, China. Tel.: + 86 13 910 770 018. E-mail address: caowc@bmi.ac.cn.

Lin Zhao, Institute of EcoHealth, School of Public Health, Cheeloo College of Medicine, Shandong University, 44 Wenhuaxi Street, Jinan 250012, China. Tel.: +86 15 169 086 516. E-mail address: zhaolin1989@sdu.edu.cn.

| Table S1. Diagnosis codes used to identify comorbidities. | |
| --- | --- |
| Comorbidities | ICD-10 code |
| Neoplasms |  |
| Cancer | C00-C75, C76-C80, C81-C85, C88, C90-C96 |
| Diseases of the blood and blood-forming organs |  |
| Anemias | D50-D64 |
| Endocrine/metabolic diseases |  |
| Disorders of the thyroid gland | E00-E07 |
| Diabetes mellitus | E10-E14 |
| Metabolic disorders | E70-E88 |
| Mental and behavioral disorders |  |
| Mental disorders | F00-F02, F05, F20-F48 |
| Nervous system diseases |  |
| Inflammatory diseases of the central nervous system | G00-G09 |
| Epilepsy | G40 |
| Disorders of the peripheral nervous system | G50-G64 |
| Diseases of the eye and adnexa |  |
| Cataract | H25-H26 |
| Circulatory system diseases |  |
| Valvular heart diseases | I05-I09, I34-I39 |
| Hypertensive diseases | I10-I15 |
| Chronic ischemic heart disease | I25 |
| Pulmonary heart disease and diseases of pulmonary circulation | I26-I28 |
| Cardiac arrhythmias | I44-I49 |
| Cerebrovascular diseases | I60-I69, G45, G46 |
| Respiratory system diseases |  |
| Chronic lower respiratory diseases | J40-J47 |
| Interstitial lung diseases | J60-J67, J84, D86 |
| Digestive system diseases |  |
| Ulcer diseases | K25-K28 |
| Noninfective enteritis and colitis | K50-K52 |
| Chronic liver diseases | K70.0-K70.3, K70.4, K70.9, K71, K72, K73, K74, K76.0, K76.6, B15.0, B16.0, B16.2, B19.0 |
| Diseases of the skin and subcutaneous tissue |  |
| Dermatitis and eczema | L20-L30 |
| Papulosquamous disorders | L40-L45 |
| Musculoskeletal/connective tissue diseases |  |
| Connective tissue diseases | M05, M06,M30-M36 |
| Intervertebral disc disorders | M50-M51 |
| Osteoporosis | M80-M82 |
| Genitourinary system diseases |  |
| Renal diseases | N00-N05, N07, N11, N14, N17-N19 |
| Disorders of prostate | N40-N42 |
| Disorders of breast | N60-N64 |
| Inflammatory diseases of female pelvic organs  tory diseases of female pelvic organs | N70-N77 |

| Table S2. Prevalence of multimorbidity at index date. | | | | | | |
| --- | --- | --- | --- | --- | --- | --- |
| Age group | Female | | Male | | Total | |
|  | Cases  n (%) | Controls  n (%) | Cases  n (%) | Controls  n (%) | Cases  n (%) | Controls  n (%) |
| 0-44 | 44 (5.50) | 38 (1.19) | 32 (3.11) | 28 (0.68) | 76 (4.16) | 66 (0.90) |
| 45-64 | 99 (23.40) | 81 (4.79) | 199 (21.72) | 181 (4.94) | 298 (22.26) | 262 (4.89) |
| 65+ | 163 (47.38) | 216 (15.70) | 312 (41.38) | 597 (19.79) | 475 (43.26) | 813 (18.51) |
| Total | 306 (19.53) | 335 (5.34) | 543 (20.13) | 806 (7.47) | 849 (19.91) | 1141 (6.69) |


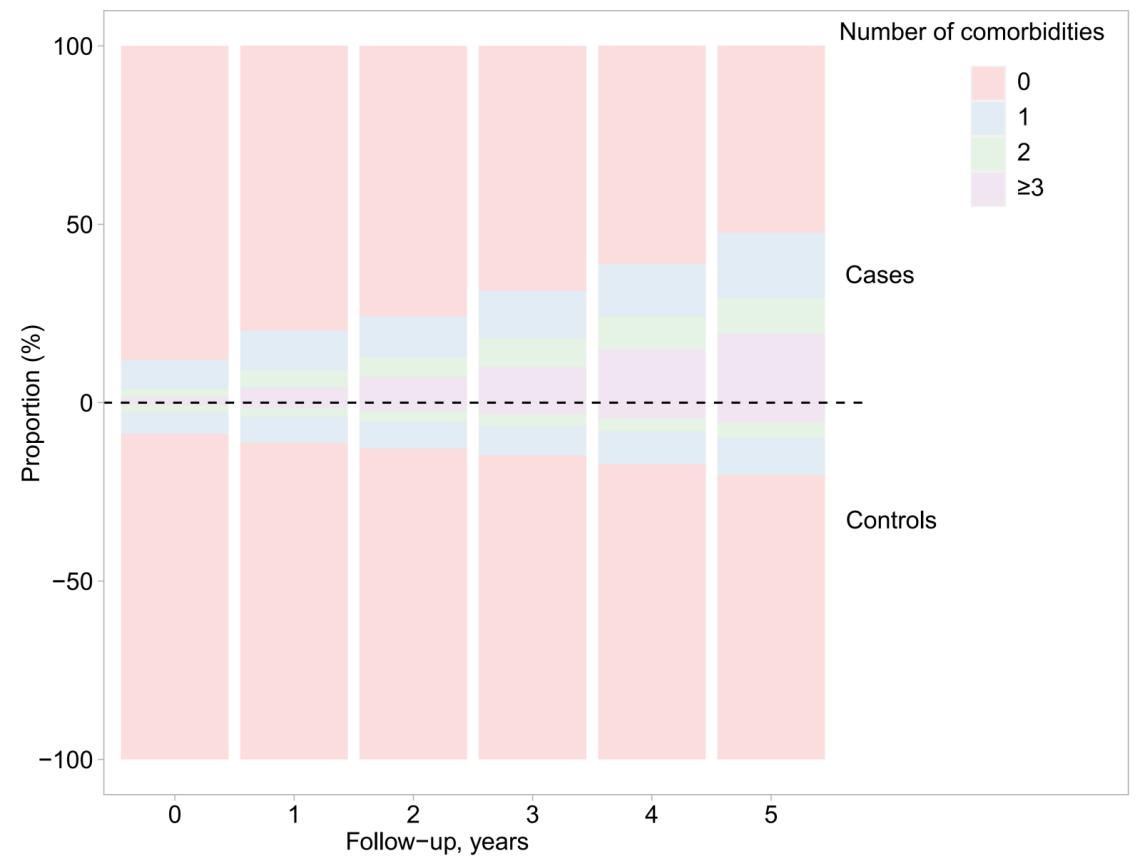


Fig. S1. Distributions of the number of comorbidities in cases and controls over time.

| Table S3. Associations between PTB and comorbidities in males. | | | | | | |
| --- | --- | --- | --- | --- | --- | --- |
| Comorbidities | Cases  n (%) | Controls  n (%) | Unadjusted | | Adjusteda | |
|  |  |  | OR (95% CI) | *** P value* | OR (95% CI) | *** P value* |
| Neoplasms |  |  |  |  |  |  |
| Cancer | 88 (3.26) | 109 (1.01) | 3.62 (2.68, 4.88) | <0.001 | 2.44 (1.71, 3.47) | <0.001 |
| Diseases of the blood and blood-forming organs |  |  |  |  |  |  |
| Anemias | 32 (1.19) | 25 (0.23) | 5.40 (3.15, 9.23) | <0.001 | 1.58 (0.83, 3.01) | 0.319 |
| Endocrine/metabolic diseases |  |  |  |  |  |  |
| Disorders of the thyroid gland | 31 (1.15) | 30 (0.28) | 4.13 (2.50, 6.83) | <0.001 | 1.80 (0.97, 3.35) | 0.136 |
| Diabetes mellitus | 269 (9.97) | 404 (3.74) | 3.07 (2.59, 3.64) | <0.001 | 2.24 (1.84, 2.72) | <0.001 |
| Metabolic disorders | 82 (3.04) | 86 (0.80) | 3.99 (2.93, 5.44) | <0.001 | 1.48 (1.01, 2.18) | 0.112 |
| Mental and behavioral disorders |  |  |  |  |  |  |
| Mental disorders | 18 (0.67) | 36 (0.33) | 2.03 (1.15, 3.61) | 0.018 | 0.65 (0.30, 1.39) | 0.420 |
| Nervous system diseases |  |  |  |  |  |  |
| Inflammatory diseases of the central nervous system | 33 (1.22) | 23 (0.21) | 5.74 (3.37, 9.77) | <0.001 | 3.03 (1.53, 6.02) | 0.011 |
| Epilepsy | 8 (0.30) | 15 (0.14) | 2.13 (0.90, 5.03) | 0.090 | 1.17 (0.44, 3.12) | 0.843 |
| Disorders of the peripheral nervous system | 34 (1.26) | 23 (0.21) | 6.50 (3.73, 11.31) | <0.001 | 2.26 (1.14, 4.49) | 0.070 |
| Diseases of the eye and adnexa |  |  |  |  |  |  |
| Cataract | 33 (1.22) | 51 (0.47) | 2.67 (1.71, 4.18) | <0.001 | 1.07 (0.60, 1.90) | 0.861 |
| Circulatory system diseases |  |  |  |  |  |  |
| Valvular heart diseases | 10 (0.37) | 17 (0.16) | 2.40 (1.09, 5.32) | 0.035 | 0.80 (0.31, 2.05) | 0.773 |
| Hypertensive diseases | 409 (15.16) | 1060 (9.82) | 1.85 (1.61, 2.12) | <0.001 | 1.11 (0.94, 1.32) | 0.388 |
| Chronic ischemic heart disease | 258 (9.56) | 465 (4.31) | 2.72 (2.28, 3.24) | <0.001 | 1.38 (1.10, 1.73) | 0.020 |
| Pulmonary heart disease and diseases of pulmonary circulation | 36 (1.33) | 26 (0.24) | 5.83 (3.48, 9.79) | <0.001 | 1.36 (0.73, 2.53) | 0.492 |
| Cardiac arrhythmias | 64 (2.37) | 81 (0.75) | 3.50 (2.47, 4.96) | <0.001 | 1.16 (0.74, 1.81) | 0.672 |
| Cerebrovascular diseases | 211 (7.82) | 407 (3.77) | 2.40 (1.99, 2.89) | <0.001 | 1.27 (1.01, 1.60) | 0.112 |
| Respiratory system diseases |  |  |  |  |  |  |
| Chronic lower respiratory diseases | 304 (11.27) | 170 (1.58) | 9.16 (7.41, 11.32) | <0.001 | 6.79 (5.40, 8.54) | <0.001 |
| Interstitial lung diseases | 33 (1.22) | 11 (0.10) | 12.00 (6.07, 23.74) | <0.001 | 5.55 (2.47, 12.46) | <0.001 |
| Digestive system diseases |  |  |  |  |  |  |
| Ulcer diseases | 15 (0.56) | 39 (0.36) | 1.57 (0.85, 2.89) | 0.155 | 0.58 (0.27, 1.26) | 0.319 |
| Noninfective enteritis and colitis | 33 (1.22) | 32 (0.30) | 4.20 (2.57, 6.86) | <0.001 | 2.52 (1.38, 4.59) | 0.014 |
| Chronic liver diseases | 15 (0.56) | 16 (0.15) | 4.03 (1.93, 8.38) | <0.001 | 2.30 (1.02, 5.18) | 0.112 |
| Diseases of the skin and subcutaneous tissue |  |  |  |  |  |  |
| Dermatitis and eczema | 12 (0.44) | 19 (0.18) | 2.53 (1.23, 5.20) | 0.015 | 1.51 (0.60, 3.81) | 0.531 |
| Papulosquamous disorders | 7 (0.26) | 12 (0.11) | 2.33 (0.92, 5.93) | 0.084 | 1.54 (0.54, 4.39) | 0.553 |
| Musculoskeletal/connective tissue diseases |  |  |  |  |  |  |
| Connective tissue diseases | 16 (0.59) | 11 (0.10) | 5.82 (2.70, 12.54) | <0.001 | 2.46 (0.98, 6.21) | 0.131 |
| Intervertebral disc disorders | 43 (1.59) | 100 (0.93) | 1.79 (1.23, 2.59) | 0.003 | 0.77 (0.48, 1.22) | 0.420 |
| Osteoporosis | 4 (0.15) | 10 (0.09) | 1.63 (0.50, 5.36) | 0.419 | 0.75 (0.18, 3.24) | 0.823 |
| Genitourinary system diseases |  |  |  |  |  |  |
| Renal diseases | 37 (1.37) | 67 (0.62) | 2.23 (1.49, 3.34) | <0.001 | 1.03 (0.63, 1.69) | 0.901 |
| Disorders of prostate | 58 (2.15) | 99 (0.92) | 2.60 (1.84, 3.69) | <0.001 | 1.05 (0.68, 1.63) | 0.861 |
| Disorders of breast | - | - | - | - | - | - |
| Inflammatory diseases of female pelvic organs | - | - | - | - | - | - |
| ^a^Adjusted for age at index date and all comorbidities.  ** *P*<0.05 adjusted for multiple testing using FDR.  FDR, false discovery rate. | | | | | | |

| Table S4. Associations between PTB and comorbidities in females. | | | | | | |
| --- | --- | --- | --- | --- | --- | --- |
| Comorbidities | Cases  n (%) | Controls  n (%) | Unadjusted | | Adjusteda | |
|  |  |  | OR (95% CI) | *** P value* | OR (95% CI) | *** P value* |
| Neoplasms |  |  |  |  |  |  |
| Cancer | 56 (3.57) | 28 (0.45) | 9.27 (5.70, 15.09) | <0.001 | 5.53 (3.20, 9.57) | <0.001 |
| Diseases of the blood and blood-forming organs |  |  |  |  |  |  |
| Anemias | 35 (2.23) | 33 (0.53) | 4.48 (2.74, 7.31) | <0.001 | 1.56 (0.83, 2.95) | 0.301 |
| Endocrine/metabolic diseases |  |  |  |  |  |  |
| Disorders of the thyroid gland | 35 (2.23) | 33 (0.53) | 4.40 (2.71, 7.14) | <0.001 | 1.88 (1.02, 3.46) | 0.111 |
| Diabetes mellitus | 124 (7.91) | 201 (3.21) | 2.85 (2.23, 3.64) | <0.001 | 1.76 (1.3, 2.39) | <0.001 |
| Metabolic disorders | 58 (3.70) | 36 (0.57) | 7.50 (4.79, 11.74) | <0.001 | 1.55 (0.84, 2.86) | 0.301 |
| Mental and behavioral disorders |  |  |  |  |  |  |
| Mental disorders | 19 (1.21) | 21 (0.34) | 3.62 (1.95, 6.73) | <0.001 | 1.31 (0.56, 3.05) | 0.617 |
| Nervous system diseases |  |  |  |  |  |  |
| Inflammatory diseases of the central nervous system | 19 (1.21) | 8 (0.13) | 9.50 (4.16, 21.70) | <0.001 | 4.85 (1.66, 14.21) | 0.012 |
| Epilepsy | 4 (0.26) | 3 (0.05) | 5.33 (1.19, 23.83) | 0.028 | 3.34 (0.66, 16.76) | 0.296 |
| Disorders of the peripheral nervous system | 15 (0.96) | 11 (0.18) | 5.80 (2.60, 12.95) | <0.001 | 1.62 (0.59, 4.49) | 0.509 |
| Diseases of the eye and adnexa |  |  |  |  |  |  |
| Cataract | 25 (1.60) | 26 (0.41) | 3.93 (2.25, 6.85) | <0.001 | 2.04 (1.01, 4.12) | 0.111 |
| Circulatory system diseases |  |  |  |  |  |  |
| Valvular heart diseases | 4 (0.26) | 3 (0.05) | 5.33 (1.19, 23.83) | 0.028 | 0.73 (0.10, 5.31) | 0.810 |
| Hypertensive diseases | 205 (13.08) | 478 (7.63) | 2.15 (1.76, 2.63) | <0.001 | 1.05 (0.81, 1.37) | 0.776 |
| Chronic ischemic heart disease | 148 (9.44) | 188 (3.00) | 4.19 (3.25, 5.39) | <0.001 | 2.11 (1.53, 2.91) | <0.001 |
| Pulmonary heart disease and diseases of pulmonary circulation | 19 (1.21) | 6 (0.10) | 12.67 (5.06, 31.72) | <0.001 | 5.81 (1.73, 19.5) | 0.012 |
| Cardiac arrhythmias | 23 (1.47) | 20 (0.32) | 5.07 (2.70, 9.53) | <0.001 | 1.49 (0.66, 3.38) | 0.509 |
| Cerebrovascular diseases | 79 (5.04) | 126 (2.01) | 2.98 (2.18, 4.08) | <0.001 | 1.39 (0.93, 2.10) | 0.248 |
| Respiratory system diseases |  |  |  |  |  |  |
| Chronic lower respiratory diseases | 158 (10.08) | 54 (0.86) | 14.70 (10.41, 20.74) | <0.001 | 9.58 (6.6, 13.92) | <0.001 |
| Interstitial lung diseases | 20 (1.28) | 2 (0.03) | 40.00 (9.35, 171.13) | <0.001 | 19.40 (4.03, 93.37) | <0.001 |
| Digestive system diseases |  |  |  |  |  |  |
| Ulcer diseases | 6 (0.38) | 4 (0.06) | 6.00 (1.69, 21.26) | 0.007 | 1.88 (0.28, 12.45) | 0.617 |
| Noninfective enteritis and colitis | 20 (1.28) | 23 (0.37) | 3.48 (1.91, 6.33) | <0.001 | 1.03 (0.45, 2.39) | 0.939 |
| Chronic liver diseases | 7 (0.45) | 6 (0.10) | 4.67 (1.57, 13.89) | 0.007 | 2.04 (0.55, 7.62) | 0.464 |
| Diseases of the skin and subcutaneous tissue |  |  |  |  |  |  |
| Dermatitis and eczema | 13 (0.83) | 16 (0.26) | 3.35 (1.59, 7.06) | 0.001 | 1.13 (0.42, 3.00) | 0.841 |
| Papulosquamous disorders | 5 (0.32) | 4 (0.06) | 5.00 (1.34, 18.62) | 0.017 | 2.07 (0.38, 11.23) | 0.551 |
| Musculoskeletal/connective tissue diseases |  |  |  |  |  |  |
| Connective tissue diseases | 19 (1.21) | 8 (0.13) | 9.50 (4.16, 21.70) | <0.001 | 6.00 (2.21, 16.29) | <0.001 |
| Intervertebral disc disorders | 34 (2.17) | 34 (0.54) | 4.14 (2.55, 6.71) | <0.001 | 2.59 (1.41, 4.74) | 0.007 |
| Osteoporosis | 10 (0.64) | 6 (0.10) | 6.67 (2.42, 18.34) | <0.001 | 1.63 (0.49, 5.41) | 0.562 |
| Genitourinary system diseases |  |  |  |  |  |  |
| Renal diseases | 19 (1.21) | 16 (0.26) | 5.15 (2.57, 10.31) | <0.001 | 1.43 (0.57, 3.61) | 0.569 |
| Disorders of prostate | - | - | - | - | - | - |
| Disorders of breast | 16 (1.02) | 24 (0.38) | 2.71 (1.43, 5.14) | 0.002 | 1.63 (0.77, 3.43) | 0.341 |
| Inflammatory diseases of female pelvic organs | 61 (3.89) | 82 (1.31) | 3.45 (2.40, 4.95) | <0.001 | 2.46 (1.64, 3.69) | <0.001 |
| ^a^Adjusted for age at index date and all comorbidities.  ** *P*<0.05 adjusted for multiple testing using FDR.  FDR, false discovery rate. | | | | | | |

| Table S5. Associations between PTB and comorbidities in patients **aged 44 years and younger.** | | | | | | |
| --- | --- | --- | --- | --- | --- | --- |
| Comorbidities | Cases  n (%) | Controls  n (%) | Unadjusted | | Adjusteda | |
|  |  |  | OR (95% CI) | *** P value* | OR (95% CI) | *** P value* |
| Neoplasms |  |  |  |  |  |  |
| Cancer | 12 (0.66) | 10 (0.14) | 5.12 (2.15, 12.19) | <0.001 | 3.66 (1.44, 9.33) | 0.027 |
| Diseases of the blood and blood-forming organs |  |  |  |  |  |  |
| Anemias | 15 (0.82) | 27 (0.37) | 2.31 (1.21, 4.43) | 0.023 | 0.89 (0.41, 1.92) | 0.818 |
| Endocrine/metabolic diseases |  |  |  |  |  |  |
| Disorders of the thyroid gland | 12 (0.66) | 12 (0.16) | 4.00 (1.8, 8.90) | 0.002 | 1.67 (0.66, 4.26) | 0.419 |
| Diabetes mellitus | 37 (2.02) | 27 (0.37) | 5.48 (3.34, 9.00) | <0.001 | 3.22 (1.82, 5.68) | <0.001 |
| Metabolic disorders | 24 (1.31) | 9 (0.12) | 10.67 (4.96, 22.95) | <0.001 | 4.90 (2.03, 11.83) | <0.001 |
| Mental and behavioral disorders |  |  |  |  |  |  |
| Mental disorders | 5 (0.27) | 18 (0.25) | 1.12 (0.40, 3.08) | 0.831 | 0.63 (0.19, 2.08) | 0.545 |
| Nervous system diseases |  |  |  |  |  |  |
| Inflammatory diseases of the central nervous system | 8 (0.44) | 3 (0.04) | 10.67 (2.83, 40.21) | <0.001 | 9.25 (2.26, 37.88) | 0.009 |
| Epilepsy | 5 (0.27) | 4 (0.05) | 5.00 (1.34, 18.62) | 0.027 | 4.07 (0.99, 16.72) | 0.138 |
| Disorders of the peripheral nervous system | 3 (0.16) | 4 (0.05) | 3.00 (0.67, 13.40) | 0.193 | 2.99 (0.57, 15.68) | 0.353 |
| Diseases of the eye and adnexa |  |  |  |  |  |  |
| Cataract | - | - | - | - | - | - |
| Circulatory system diseases |  |  |  |  |  |  |
| Valvular heart diseases | - | - | - | - | - | - |
| Hypertensive diseases | 31 (1.70) | 49 (0.67) | 2.64 (1.66, 4.19) | <0.001 | 1.39 (0.78, 2.46) | 0.419 |
| Chronic ischemic heart disease | 16 (0.88) | 16 (0.22) | 4.00 (2.00, 8.00) | <0.001 | 1.21 (0.49, 3.01) | 0.762 |
| Pulmonary heart disease and diseases of pulmonary circulation | 1 (0.05) | 1 (0.01) | 4.00 (0.25, 63.95) | 0.384 | 4.00 (0.25, 63.95) | 0.458 |
| Cardiac arrhythmias | 10 (0.55) | 3 (0.04) | 13.33 (3.67, 48.45) | <0.001 | 6.63 (1.62, 27.17) | 0.030 |
| Cerebrovascular diseases | 15 (0.82) | 12 (0.16) | 5.00 (2.34, 10.68) | <0.001 | 1.92 (0.72, 5.10) | 0.353 |
| Respiratory system diseases |  |  |  |  |  |  |
| Chronic lower respiratory diseases | 63 (3.45) | 46 (0.63) | 6.00 (4.03, 8.93) | <0.001 | 4.76 (3.12, 7.26) | <0.001 |
| Interstitial lung diseases | - | - | - | - | - | - |
| Digestive system diseases |  |  |  |  |  |  |
| Ulcer diseases | 3 (0.16) | 5 (0.07) | 2.40 (0.57, 10.04) | 0.284 | 1.27 (0.23, 7.05) | 0.818 |
| Noninfective enteritis and colitis | 7 (0.38) | 20 (0.27) | 1.40 (0.59, 3.31) | 0.500 | 0.58 (0.19, 1.77) | 0.458 |
| Chronic liver diseases | 3 (0.16) | 2 (0.03) | 6.00 (1.00, 35.91) | 0.071 | 6.44 (1.07, 38.95) | 0.126 |
| Diseases of the skin and subcutaneous tissue |  |  |  |  |  |  |
| Dermatitis and eczema | 5 (0.27) | 16 (0.22) | 1.25 (0.46, 3.41) | 0.716 | 0.74 (0.24, 2.33) | 0.717 |
| Papulosquamous disorders | 3 (0.16) | 4 (0.05) | 3.00 (0.67, 13.40) | 0.193 | 2.69 (0.46, 15.71) | 0.419 |
| Musculoskeletal/connective tissue diseases |  |  |  |  |  |  |
| Connective tissue diseases | 4 (0.22) | 3 (0.04) | 5.33 (1.19, 23.83) | 0.044 | 4.77 (0.92, 24.65) | 0.140 |
| Intervertebral disc disorders | 15 (0.82) | 12 (0.16) | 5.00 (2.34, 10.68) | <0.001 | 3.61 (1.57, 8.28) | 0.009 |
| Osteoporosis | 3 (0.16) | 2 (0.03) | 6.00 (1.00, 35.91) | 0.071 | 4.10 (0.52, 32.22) | 0.353 |
| Genitourinary system diseases |  |  |  |  |  |  |
| Renal diseases | 14 (0.77) | 23 (0.31) | 2.47 (1.26, 4.85) | 0.017 | 2.05 (0.96, 4.36) | 0.140 |
| Disorders of prostate | 1 (0.05) | 3 (0.04) | 1.33 (0.14, 12.82) | 0.831 | 0.98 (0.09, 11.04) | 0.984 |
| Disorders of breast | 10 (0.55) | 15 (0.21) | 2.74 (1.21, 6.20) | 0.027 | 1.50 (0.6, 3.76) | 0.494 |
| Inflammatory diseases of female pelvic organs | 46 (2.52) | 67 (0.92) | 3.25 (2.14, 4.93) | <0.001 | 2.60 (1.66, 4.06) | <0.001 |
| ^a^Adjusted for sex and all comorbidities.  ** *P*<0.05 adjusted for multiple testing using FDR.  FDR, false discovery rate. | | | | | | |

| Table S6. Associations between PTB and comorbidities in patients aged 45 to 64. | | | | | | |
| --- | --- | --- | --- | --- | --- | --- |
| Comorbidities | Cases  n (%) | Controls  n (%) | Unadjusted | | Adjusteda | |
|  |  |  | OR (95% CI) | *** P value* | OR (95% CI) | *** P value* |
| Neoplasms |  |  |  |  |  |  |
| Cancer | 70 (5.23) | 44 (0.82) | 7.93 (5.23, 12.04) | <0.001 | 5.52 (3.31, 9.21) | <0.001 |
| Diseases of the blood and blood-forming organs |  |  |  |  |  |  |
| Anemias | 27 (2.02) | 6 (0.11) | 18.00 (7.43, 43.60) | <0.001 | 5.68 (1.92, 16.78) | 0.010 |
| Endocrine/metabolic diseases |  |  |  |  |  |  |
| Disorders of the thyroid gland | 35 (2.61) | 22 (0.41) | 6.80 (3.92, 11.79) | <0.001 | 2.98 (1.41, 6.31) | 0.017 |
| Diabetes mellitus | 187 (13.97) | 179 (3.34) | 4.89 (3.91, 6.11) | <0.001 | 3.56 (2.73, 4.64) | <0.001 |
| Metabolic disorders | 53 (3.96) | 31 (0.58) | 7.75 (4.84, 12.42) | <0.001 | 1.63 (0.84, 3.16) | 0.232 |
| Mental and behavioral disorders |  |  |  |  |  |  |
| Mental disorders | 13 (0.97) | 14 (0.26) | 3.71 (1.75, 7.90) | 0.001 | 1.05 (0.36, 3.06) | 0.959 |
| Nervous system diseases |  |  |  |  |  |  |
| Inflammatory diseases of the central nervous system | 21 (1.57) | 11 (0.21) | 7.64 (3.68, 15.84) | <0.001 | 3.95 (1.40, 11.13) | 0.034 |
| Epilepsy | 5 (0.37) | 6 (0.11) | 3.33 (1.02, 10.92) | 0.052 | 0.96 (0.21, 4.40) | 0.959 |
| Disorders of the peripheral nervous system | 27 (2.02) | 11 (0.21) | 11.51 (5.4, 24.52) | <0.001 | 2.35 (0.90, 6.13) | 0.158 |
| Diseases of the eye and adnexa |  |  |  |  |  |  |
| Cataract | 14 (1.05) | 7 (0.13) | 8.00 (3.23, 19.82) | <0.001 | 3.26 (1.00, 10.69) | 0.120 |
| Circulatory system diseases |  |  |  |  |  |  |
| Valvular heart diseases | 4 (0.30) | 5 (0.09) | 3.20 (0.86, 11.92) | 0.089 | 0.25 (0.03, 1.89) | 0.267 |
| Hypertensive diseases | 216 (16.13) | 449 (8.38) | 2.17 (1.82, 2.60) | <0.001 | 1.11 (0.87, 1.41) | 0.479 |
| Chronic ischemic heart disease | 120 (8.96) | 122 (2.28) | 4.47 (3.41, 5.85) | <0.001 | 2.24 (1.58, 3.19) | <0.001 |
| Pulmonary heart disease and diseases of pulmonary circulation | 14 (1.05) | 3 (0.06) | 18.67 (5.36, 64.95) | <0.001 | 6.10 (1.32, 28.27) | 0.070 |
| Cardiac arrhythmias | 18 (1.34) | 14 (0.26) | 5.67 (2.72, 11.82) | <0.001 | 1.57 (0.59, 4.19) | 0.462 |
| Cerebrovascular diseases | 74 (5.53) | 105 (1.96) | 3.12 (2.28, 4.29) | <0.001 | 1.43 (0.95, 2.16) | 0.161 |
| Respiratory system diseases |  |  |  |  |  |  |
| Chronic lower respiratory diseases | 138 (10.31) | 33 (0.62) | 21.39 (13.96, 32.77) | <0.001 | 15.99 (10.10, 25.31) | <0.001 |
| Interstitial lung diseases | 18 (1.34) | 4 (0.07) | 18.00 (6.09, 53.19) | <0.001 | 12.19 (3.11, 47.74) | <0.001 |
| Digestive system diseases |  |  |  |  |  |  |
| Ulcer diseases | 11 (0.82) | 7 (0.13) | 6.99 (2.57, 18.98) | <0.001 | 3.47 (0.97, 12.43) | 0.120 |
| Noninfective enteritis and colitis | 20 (1.49) | 14 (0.26) | 6.00 (2.98, 12.08) | <0.001 | 2.61 (0.98, 6.94) | 0.120 |
| Chronic liver diseases | 16 (1.19) | 8 (0.15) | 8.84 (3.63, 21.53) | <0.001 | 3.30 (1.15, 9.47) | 0.081 |
| Diseases of the skin and subcutaneous tissue |  |  |  |  |  |  |
| Dermatitis and eczema | 11 (0.82) | 5 (0.09) | 8.8 (3.06, 25.33) | <0.001 | 2.96 (0.58, 14.98) | 0.271 |
| Papulosquamous disorders | 8 (0.60) | 3 (0.06) | 10.67 (2.83, 40.21) | <0.001 | 3.16 (0.69, 14.54) | 0.232 |
| Musculoskeletal/connective tissue diseases |  |  |  |  |  |  |
| Connective tissue diseases | 13 (0.97) | 6 (0.11) | 8.67 (3.29, 22.80) | <0.001 | 3.82 (1.11, 13.13) | 0.093 |
| Intervertebral disc disorders | 24 (1.79) | 39 (0.73) | 2.61 (1.54, 4.42) | <0.001 | 0.75 (0.37, 1.52) | 0.497 |
| Osteoporosis | 3 (0.22) | 5 (0.09) | 2.40 (0.57, 10.04) | 0.231 | 0.28 (0.03, 3.02) | 0.381 |
| Genitourinary system diseases |  |  |  |  |  |  |
| Renal diseases | 24 (1.79) | 14 (0.26) | 6.86 (3.55, 13.26) | <0.001 | 1.64 (0.67, 3.99) | 0.379 |
| Disorders of prostate | 13 (0.97) | 8 (0.15) | 6.5 (2.69, 15.68) | <0.001 | 1.30 (0.38, 4.38) | 0.750 |
| Disorders of breast | 4 (0.30) | 7 (0.13) | 2.29 (0.67, 7.81) | 0.193 | 1.17 (0.26, 5.17) | 0.898 |
| Inflammatory diseases of female pelvic organs | 13 (0.97) | 9 (0.17) | 6.25 (2.58, 15.12) | <0.001 | 2.67 (0.83, 8.59) | 0.175 |
| ^a^Adjusted for sex and all comorbidities.  ** *P*<0.05 adjusted for multiple testing using FDR.  FDR, false discovery rate. | | | | | | |

| Table S7. Associations between PTB and comorbidities in patients over 65. | | | | | | |
| --- | --- | --- | --- | --- | --- | --- |
| Comorbidities | Cases  n (%) | Controls  n (%) | Unadjusted | | Adjusteda | |
|  |  |  | OR (95% CI) | *** P value* | OR (95% CI) | *** P value* |
| Neoplasms |  |  |  |  |  |  |
| Cancer | 62 (5.65) | 83 (1.89) | 3.25 (2.30, 4.59) | <0.001 | 2.13 (1.41, 3.21) | <0.001 |
| Diseases of the blood and blood-forming organs |  |  |  |  |  |  |
| Anemias | 25 (2.28) | 25 (0.57) | 4.19 (2.37, 7.39) | <0.001 | 1.51 (0.74, 3.09) | 0.462 |
| Endocrine/metabolic diseases |  |  |  |  |  |  |
| Disorders of the thyroid gland | 19 (1.73) | 29 (0.66) | 2.62 (1.47, 4.67) | 0.002 | 0.98 (0.46, 2.06) | 0.965 |
| Diabetes mellitus | 169 (15.39) | 399 (9.08) | 1.87 (1.53, 2.28) | <0.001 | 1.29 (1.02, 1.63) | 0.113 |
| Metabolic disorders | 63 (5.74) | 82 (1.87) | 3.24 (2.31, 4.55) | <0.001 | 1.19 (0.77, 1.83) | 0.614 |
| Mental and behavioral disorders |  |  |  |  |  |  |
| Mental disorders | 19 (1.73) | 25 (0.57) | 3.04 (1.67, 5.52) | <0.001 | 0.98 (0.44, 2.21) | 0.965 |
| Nervous system diseases |  |  |  |  |  |  |
| Inflammatory diseases of the central nervous system | 23 (2.09) | 17 (0.39) | 5.41 (2.89, 10.13) | <0.001 | 2.19 (0.92, 5.25) | 0.182 |
| Epilepsy | 2 (0.18) | 8 (0.18) | 1.00 (0.21, 4.71) | 1.000 | 0.62 (0.12, 3.14) | 0.676 |
| Disorders of the peripheral nervous system | 19 (1.73) | 19 (0.43) | 4.25 (2.20, 8.21) | <0.001 | 2.08 (0.88, 4.92) | 0.208 |
| Diseases of the eye and adnexa |  |  |  |  |  |  |
| Cataract | 44 (4.01) | 68 (1.55) | 2.67 (1.81, 3.93) | <0.001 | 1.57 (0.97, 2.54) | 0.177 |
| Circulatory system diseases |  |  |  |  |  |  |
| Valvular heart diseases | 10 (0.91) | 13 (0.30) | 3.19 (1.37, 7.42) | 0.010 | 1.48 (0.54, 4.06) | 0.614 |
| Hypertensive diseases | 367 (33.42) | 1040 (23.68) | 1.73 (1.48, 2.02) | <0.001 | 1.06 (0.88, 1.28) | 0.676 |
| Chronic ischemic heart disease | 270 (24.59) | 515 (11.73) | 2.66 (2.23, 3.16) | <0.001 | 1.51 (1.20, 1.90) | <0.001 |
| Pulmonary heart disease and diseases of pulmonary circulation | 40 (3.64) | 28 (0.64) | 6.00 (3.66, 9.84) | <0.001 | 1.42 (0.78, 2.58) | 0.462 |
| Cardiac arrhythmias | 59 (5.37) | 84 (1.91) | 3.09 (2.17, 4.40) | <0.001 | 0.96 (0.61, 1.52) | 0.919 |
| Cerebrovascular diseases | 201 (18.31) | 416 (9.47) | 2.25 (1.86, 2.73) | <0.001 | 1.31 (1.03, 1.66) | 0.101 |
| Respiratory system diseases |  |  |  |  |  |  |
| Chronic lower respiratory diseases | 261 (23.77) | 145 (3.30) | 9.65 (7.64, 12.20) | <0.001 | 6.67 (5.16, 8.62) | <0.001 |
| Interstitial lung diseases | 31 (2.82) | 9 (0.20) | 13.78 (6.56, 28.94) | <0.001 | 5.76 (2.42, 13.71) | <0.001 |
| Digestive system diseases |  |  |  |  |  |  |
| Ulcer diseases | 7 (0.64) | 31 (0.71) | 0.90 (0.39, 2.07) | 0.861 | 0.27 (0.10, 0.74) | 0.055 |
| Noninfective enteritis and colitis | 26 (2.37) | 21 (0.48) | 4.95 (2.79, 8.80) | <0.001 | 2.79 (1.34, 5.82) | 0.036 |
| Chronic liver diseases | 3 (0.27) | 12 (0.27) | 1.00 (0.28, 3.62) | 1.000 | 0.60 (0.14, 2.52) | 0.635 |
| Diseases of the skin and subcutaneous tissue |  |  |  |  |  |  |
| Dermatitis and eczema | 9 (0.82) | 14 (0.32) | 2.65 (1.13, 6.22) | 0.034 | 1.68 (0.59, 4.78) | 0.527 |
| Papulosquamous disorders | 1 (0.09) | 9 (0.20) | 0.44 (0.06, 3.51) | 0.510 | 0.09 (0.01, 1.16) | 0.177 |
| Genitourinary system diseases |  |  |  |  |  |  |
| Connective tissue diseases | 18 (1.64) | 10 (0.23) | 7.20 (3.32, 15.60) | <0.001 | 3.16 (1.24, 8.05) | 0.069 |
| Intervertebral disc disorders | 38 (3.46) | 83 (1.89) | 1.90 (1.28, 2.83) | 0.003 | 0.94 (0.57, 1.54) | 0.889 |
| Osteoporosis | 8 (0.73) | 9 (0.20) | 3.78 (1.41, 10.12) | 0.011 | 1.85 (0.56, 6.14) | 0.522 |
| Genitourinary system diseases |  |  |  |  |  |  |
| Renal diseases | 18 (1.64) | 46 (1.05) | 1.58 (0.91, 2.76) | 0.129 | 0.61 (0.30, 1.23) | 0.334 |
| Disorders of prostate | 44 (4.01) | 88 (2.00) | 2.21 (1.50, 3.27) | <0.001 | 1.11 (0.68, 1.81) | 0.783 |
| Disorders of breast | 2 (0.18) | 2 (0.05) | 4.00 (0.56, 28.4) | 0.199 | 6.76 (0.80, 57.05) | 0.182 |
| Inflammatory diseases of female pelvic organs | 2 (0.18) | 6 (0.14) | 1.33 (0.27, 6.61) | 0.806 | 0.44 (0.07, 2.89) | 0.593 |
| ^a^Adjusted for sex and all comorbidities.  ** *P*<0.05 adjusted for multiple testing using FDR.  FDR, false discovery rate. | | | | | | |

| Table S8. Centrality analysis of comorbidity network in PTB patients. | | | |
| --- | --- | --- | --- |
| Comorbidities | Degree | Betweenness | Closeness |
| Disorders of the peripheral nervous system | 10.000 | 0.000 | 0.017 |
| Metabolic disorders | 12.000 | 0.000 | 0.018 |
| Disorders of the thyroid gland | 12.000 | 0.000 | 0.021 |
| Inflammatory diseases of the central nervous system | 8.000 | 0.000 | 0.017 |
| Noninfective enteritis and colitis | 10.000 | 0.000 | 0.019 |
| Cerebrovascular diseases | 12.000 | 7.000 | 0.023 |
| Diabetes mellitus | 12.000 | 14.000 | 0.027 |
| Cancer | 11.000 | 10.000 | 0.026 |
| Interstitial lung diseases | 9.000 | 0.000 | 0.020 |
| Chronic ischemic heart disease | 11.000 | 1.000 | 0.022 |
| Chronic lower respiratory diseases | 12.000 | 21.000 | 0.027 |
| Chronic liver diseases | 5.000 | 0.000 | 0.015 |
| Connective tissue diseases | 6.000 | 0.000 | 0.020 |
| Inflammatory diseases of female pelvic organs | 4.000 | 0.000 | 0.021 |

| Table S9. Data in the form of comorbidity network in PTB patients. | | | | |
| --- | --- | --- | --- | --- |
| ID | Disease name | Disease name | OER (95% CI) | *** P value* |
| 1 | Disorders of the peripheral nervous system | Inflammatory diseases of the central nervous system | 25.11(22.03, 28.62) | <0.001 |
| 2 | Connective tissue diseases | Interstitial lung diseases | 18.39(14.39, 23.51) | <0.001 |
| 3 | Chronic liver diseases | Disorders of the peripheral nervous system | 11.87(6.17, 22.84) | <0.001 |
| 4 | Noninfective enteritis and colitis | Inflammatory diseases of the central nervous system | 10.83(8.18, 14.33) | <0.001 |
| 5 | Chronic liver diseases | Cancer | 9.42(7.12, 12.47) | <0.001 |
| 6 | Connective tissue diseases | Metabolic disorders | 8.70(7.15, 10.58) | <0.001 |
| 7 | Disorders of the peripheral nervous system | Metabolic disorders | 8.70(7.56, 10.01) | <0.001 |
| 8 | Disorders of the peripheral nervous system | Diabetes mellitus | 7.09(6.67, 7.54) | <0.001 |
| 9 | Inflammatory diseases of the central nervous system | Metabolic disorders | 7.03(5.97, 8.28) | <0.001 |
| 10 | Disorders of the peripheral nervous system | Disorders of the thyroid gland | 6.59(4.45, 9.75) | <0.001 |
| 11 | Inflammatory diseases of female pelvic organs | Disorders of the thyroid gland | 6.36(4.59, 8.82) | <0.001 |
| 12 | Noninfective enteritis and colitis | Disorders of the thyroid gland | 6.10(4.12, 9.03) | <0.001 |
| 13 | Cerebrovascular diseases | Inflammatory diseases of the central nervous system | 5.94(5.41, 6.52) | <0.001 |
| 14 | Chronic liver diseases | Disorders of the thyroid gland | 5.87(2.20, 15.65) | <0.001 |
| 15 | Disorders of the thyroid gland | Cancer | 5.83(5.02, 6.78) | <0.001 |
| 16 | Inflammatory diseases of the central nervous system | Diabetes mellitus | 5.43(5.04, 5.85) | <0.001 |
| 17 | Noninfective enteritis and colitis | Cerebrovascular diseases | 5.27(4.76, 5.84) | <0.001 |
| 18 | Cerebrovascular diseases | Metabolic disorders | 5.25(5.05, 5.46) | <0.001 |
| 19 | Cerebrovascular diseases | Disorders of the peripheral nervous system | 5.10(4.55, 5.72) | <0.001 |
| 20 | Metabolic disorders | Disorders of the thyroid gland | 5.08(4.25, 6.07) | <0.001 |
| 21 | Interstitial lung diseases | Chronic lower respiratory diseases | 4.88(4.55, 5.23) | <0.001 |
| 22 | Cerebrovascular diseases | Chronic ischemic heart disease | 4.85(4.78, 4.92) | <0.001 |
| 23 | Disorders of the peripheral nervous system | Cancer | 4.84(3.79, 6.18) | <0.001 |
| 24 | Interstitial lung diseases | Metabolic disorders | 4.60(3.60, 5.88) | <0.001 |
| 25 | Noninfective enteritis and colitis | Chronic ischemic heart disease | 4.56(4.19, 4.96) | <0.001 |
| 26 | Noninfective enteritis and colitis | Chronic lower respiratory diseases | 4.35(4.02, 4.70) | <0.001 |
| 27 | Metabolic disorders | Diabetes mellitus | 3.88(3.73, 4.03) | <0.001 |
| 28 | Chronic ischemic heart disease | Inflammatory diseases of the central nervous system | 3.84(3.46, 4.26) | <0.001 |
| 29 | Inflammatory diseases of the central nervous system | Disorders of the thyroid gland | 3.73(1.94, 7.17) | <0.001 |
| 30 | Interstitial lung diseases | Disorders of the thyroid gland | 3.66(1.90, 7.03) | <0.001 |
| 31 | Chronic ischemic heart disease | Metabolic disorders | 3.60(3.46, 3.75) | <0.001 |
| 32 | Diabetes mellitus | Disorders of the thyroid gland | 3.45(3.14, 3.79) | <0.001 |
| 33 | Chronic lower respiratory diseases | Metabolic disorders | 3.30(3.17, 3.43) | <0.001 |
| 34 | Noninfective enteritis and colitis | Disorders of the peripheral nervous system | 3.28(1.23, 8.74) | 0.022 |
| 35 | Metabolic disorders | Cancer | 3.17(2.78, 3.61) | <0.001 |
| 36 | Interstitial lung diseases | Chronic ischemic heart disease | 3.17(2.81, 3.58) | <0.001 |
| 37 | Chronic lower respiratory diseases | Chronic ischemic heart disease | 3.12(3.08, 3.16) | <0.001 |
| 38 | Chronic ischemic heart disease | Diabetes mellitus | 3.07(3.02, 3.12) | <0.001 |
| 39 | Interstitial lung diseases | Cerebrovascular diseases | 3.05(2.55, 3.64) | <0.001 |
| 40 | Noninfective enteritis and colitis | Interstitial lung diseases | 3.04(1.14, 8.10) | 0.033 |
| 41 | Chronic liver diseases | Diabetes mellitus | 2.96(2.14, 4.10) | <0.001 |
| 42 | Cerebrovascular diseases | Diabetes mellitus | 2.96(2.89, 3.03) | <0.001 |
| 43 | Connective tissue diseases | Cerebrovascular diseases | 2.94(2.22, 3.89) | <0.001 |
| 44 | Chronic lower respiratory diseases | Cerebrovascular diseases | 2.90(2.84, 2.96) | <0.001 |
| 45 | Noninfective enteritis and colitis | Metabolic disorders | 2.87(1.94, 4.25) | <0.001 |
| 46 | Chronic ischemic heart disease | Disorders of the peripheral nervous system | 2.79(2.40, 3.24) | <0.001 |
| 47 | Chronic lower respiratory diseases | Cancer | 2.76(2.64, 2.89) | <0.001 |
| 48 | Chronic ischemic heart disease | Disorders of the thyroid gland | 2.71(2.42, 3.04) | <0.001 |
| 49 | Connective tissue diseases | Chronic ischemic heart disease | 2.70(2.17, 3.36) | <0.001 |
| 50 | Chronic lower respiratory diseases | Disorders of the thyroid gland | 2.52(2.26, 2.81) | <0.001 |
| 51 | Noninfective enteritis and colitis | Diabetes mellitus | 2.46(2.09, 2.90) | <0.001 |
| 52 | Inflammatory diseases of female pelvic organs | Cancer | 2.43(1.64, 3.60) | <0.001 |
| 53 | Chronic lower respiratory diseases | Disorders of the peripheral nervous system | 2.26(1.92, 2.66) | <0.001 |
| 54 | Noninfective enteritis and colitis | Cancer | 2.24(1.37, 3.66) | 0.001 |
| 55 | Interstitial lung diseases | Cancer | 2.24(1.37, 3.66) | 0.001 |
| 56 | Chronic ischemic heart disease | Cancer | 2.19(2.05, 2.34) | <0.001 |
| 57 | Connective tissue diseases | Diabetes mellitus | 2.17(1.64, 2.87) | <0.001 |
| 58 | Chronic lower respiratory diseases | Inflammatory diseases of the central nervous system | 2.13(1.81, 2.51) | <0.001 |
| 59 | Connective tissue diseases | Chronic lower respiratory diseases | 2.11(1.65, 2.69) | <0.001 |
| 60 | Interstitial lung diseases | Diabetes mellitus | 2.05(1.69, 2.49) | <0.001 |
| 61 | Chronic liver diseases | Cerebrovascular diseases | 2.01(1.05, 3.86) | 0.044 |
| 62 | Inflammatory diseases of female pelvic organs | Metabolic disorders | 2.00(1.23, 3.26) | 0.007 |
| 63 | Cerebrovascular diseases | Cancer | 1.74(1.55, 1.95) | <0.001 |
| 64 | Chronic lower respiratory diseases | Diabetes mellitus | 1.69(1.65, 1.74) | <0.001 |
| 65 | Diabetes mellitus | Cancer | 1.66(1.52, 1.81) | <0.001 |
| 66 | Cerebrovascular diseases | Disorders of the thyroid gland | 1.56(1.18, 2.06) | 0.003 |
| 67 | Inflammatory diseases of female pelvic organs | Chronic lower respiratory diseases | 1.51(1.24, 1.84) | <0.001 |
| *** P*<0.05 adjusted for multiple testing using FDR.  FDR, false discovery rate.  OER, observed-to-expected ratio. | | | | |
